# Neutron source strength measurements for Varian, Siemens, Elekta, and General Electric linear accelerators

**DOI:** 10.1120/jacmp.v4i3.2514

**Published:** 2003-06-01

**Authors:** David S. Followill, Marilyn S. Stovall, Stephen F. Kry, Geoffrey S. Ibbott

**Affiliations:** ^1^ Department of Radiation Physics The University of Texas M. D. Anderson Cancer Center 1515 Holcombe Boulevard Houston Texas 77030

**Keywords:** neutron source strength, shielding, photoneutron

## Abstract

The shielding calculations for high energy (> 10 MV) linear accelerators must include the photoneutron production within the head of the accelerator. Procedures have been described to calculate the treatment room door shielding based on the neutron source strength (*Q* value) for a specific accelerator and energy combination. Unfortunately, there is currently little data in the literature stating the neutron source strengths for the most widely used linear accelerators. In this study, the neutron fluence for 36 linear accelerators, including models from Varian, Siemens, Elekta/Philips, and General Electric, was measured using gold‐foil activation. Several of the models and energy combinations had multiple measurements. The neutron fluence measured in the patient plane was independent of the surface area of the room, suggesting that neutron fluence is more dependent on the direct neutron fluence from the head of the accelerator than from room scatter. Neutron source strength, *Q*, was determined from the measured neutron fluences. As expected, *Q* increased with increasing photon energy. The *Q* values ranged from 0.02 for a 10 MV beam to $1.44(\times 10^{12})$ neutrons per photon Gy for a 25 MV beam. The most comprehensive set of neutron source strength values, *Q*, for the current accelerators in clinical use are presented for use in calculating room shielding.

PACS number(s): 87.53.–j, 87.52.–g

## INTRODUCTION

In most radiotherapy facilities, the older low‐energy linear accelerators (linacs) are being replaced with new dual‐energy linacs with photon beams ≥ 10 MV; as well, new treatment rooms are being built to accommodate the new dual‐energy linacs. Thus, the number of linacs with high‐energy photon beams (≥ 10 MV) is increasing. These new linacs have the capacity to produce photoneutrons in the target, flattening filters and collimating devices if operated at energies above 10 MeV The neutron component in treatment rooms where photon energies ≥ 15 MV are produced is significant and, as such, extra shielding is required. Measurements by Palta *et al.* ^1^ have shown a six‐fold increase in the neutron dose around two Siemens Mevatron 77 accelerators (Siemens Medical Solutions, Concord, CA) as the photon energy was increased from 15 to 18 MV

The data required to perform the shielding calculations for the neutrons, especially the shielding in the treatment door, are lacking. National Council on Radiation Protection and Measurements (NCRP) Reports 51 and 79 addressed the shielding requirements for the higher x‐ray energies and photoneutrons, respectively; however, they were published over 16 years ago and have been superceded by new research and concepts.^2,^, ^3^ McGinley^4^ described a method to calculate the amount of shielding needed in the treatment door to account for photoneutron production using what he referred to as the neutron source strength for a specific linac. McGinley provided a table of neutron source strengths; however, the table lacked values for the variety of newer linac models produced by current manufacturers.

Both NCRP Report 79 and McGinley's research described a method to determine the total neutron fluence ($n/{\text{cm}}^{2}$) per unit x‐ray dose at isocenter produced by several different linacs.^3,^, ^4^ The total neutron fluence is given by the sum of the direct, scatter, and thermal neutron fluences:

(1) $$\Phi_{\text{total}}=\Phi_{\text{dir}}+\Phi_{\text{sc}}+\Phi_{\text{th}}.$$

A Monte Carlo analysis by McCall *et al.* ^5,^, ^6^ of the effects of a concrete room on the neutron spectrum indicated that each of the neutron fluence components could be determined using the following empirical relationships:

(2) $$\Phi_{\text{total}}=\Phi_{\text{dir}}+\Phi_{\text{sc}}+\Phi_{\text{th}}=(aQ/4\pi d^{2})+(5.4aQ/S)+(1.26Q/S).$$

Equation (2) can be broken down into the following three components:

(3) $$\begin{array}{l} \Phi_{\text{dir}}=\left(aQ/4\pi d^{2}\right),\\ \Phi_{\text{sc}}=\left(5.4aQ/S\right),\\ \Phi_{\text{th}}=\left(1.26Q/S\right). \end{array}$$

The quantity “*a*” is the transmission factor for neutrons that penetrate the linac head shielding. The transmission factor “*a*” has a value of 1.0 for lead and 0.85 for tungsten. The quantity “*S*” is the treatment‐room surface area in square centimeters, and “*Q*” is the neutron source strength in neutrons from the head of the treatment unit per x‐ray dose (Gy) delivered at isocenter. The quantity “*d*” is the distance (cm) from the target to the point where the direct fluence is evaluated. From the relationship in Eq. (2) the total neutron fluence, $\Phi_{\text{total}}$, can be determined. The total neutron fluence is then used to estimate the capture gamma dose at the maze entrance per x‐ray dose delivered at isocenter.^6^ Once the capture gamma dose is known at the maze entrance as well as the scattered and transmitted x‐ray dose, the lead thickness of the maze door shielding can be calculated.

Neutron source strength values (*Q* values) have been published for only a limited number of linac models and x‐ray energies. The neutron source strengths for many of the current and newer linacs are not available in the literature.^4,^, ^7,^ ^–^ ^12^ The work presented here reports the *Q* values for 36 modern linacs representing 12 different combinations of linac models and x‐ray energies.

## MATERIALS AND METHODS

**Figure 1:**
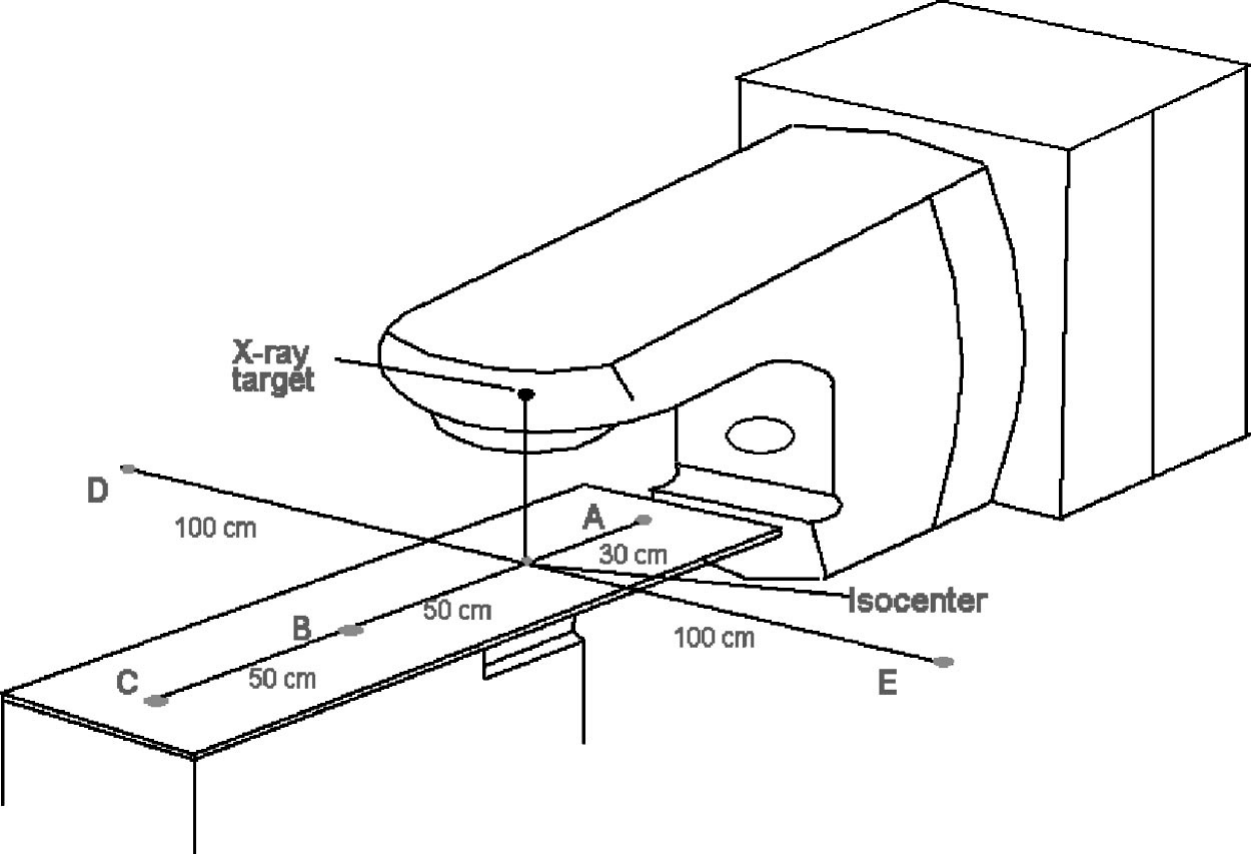
Location of the gold foils and moderators (points A‐E) for the neutron fluence measurements around the linear accelerators.

Estimates of total neutron fluence in and around a radiation therapy treatment room require that measurements of fast and thermal neutrons be made. For this work, the fast neutron fluence in a number of treatment rooms was measured using the gold‐foil activation technique outlined previously in the literature.^1,^, ^13^ Neutron measurements were made for Varian models 2100C, 2300CD, and 2500 (Varian Associates, Palo Alto, CA), Siemens models Primus, KD, MD, and MD2 (Siemens Medical Solutions, Concord, CA). Elekta models SL20 and SL25 (Elekta Oncology Systems, Norcross, GA), and General Electric model Saturn 43 (General Electric Medical Systems, Buc, France). Briefly, the gold foils (~2 cm diameter ×~0.025 mm thick) were weighed to subsequently account for the different foil sizes used and were then placed inside neutron polyethylene moderators (Reactor Experiments, Sunnyvale, CA). The moderators were used to thermalize the fast neutrons such that the gold foils were exposed only to thermal neutrons. Gold foils have a high cross section of interaction for thermal neutron energies and are subsequently activated by the neutrons. The moderators also had a cadmium covering, which absorbs incident thermal neutrons so that only thermalized fast neutrons reached the gold foils. The moderators were placed on the treatment couch at various locations in the patient plane, as shown in Fig. 1. The foils were located in a plane perpendicular to the central axis of the beam located 100 cm from the target. The center of the foil was considered the point of measurement.

The gold foils were exposed to the neutron fluences generated by x‐ray beams of energies ranging from 10 to 25 MV. The accelerators used and the photon energies available from each are listed in Table I. The neutron fluence measured from an accelerator varies with the collimator setting. The secondary collimators were set to a 20−cm×20−cm opening for most measurements, which was used to represent a clinically relevant jaw setting. It has been shown however, that as the jaw setting is reduced to a 0−cm×0−cm opening, the neutron production increases.^1^ The collimator setting for the intensity modulated radiation therapy setups was approximately 4 cm×4 cm. A total of 30 photon Gy at the depth of maximum dose was delivered to isocenter for all beams.

**Table I:** Neutron source strength (*Q*) values for various linac model and energy combinations.

Manufacturer	Model	Nominal MV	*Q* values ($\times 10^{12}$ neutrons per Gy)	Std. Deviation ($\times 10^{12}$)	No. of Linac s	Published *Q* values ($\times 10^{12}$)
Varian	1800	10				0.06
Varian	1800	15				0.76
Varian	1800	18				1.22
Varian	2100C	18	0.96	0.11	17
Varian	2100C^b^	18	0.87		1
Varian	2300CD	18	0.95	0.03	2
Varian	2500	24	0.77		1
Siemens	MD2	10	0.08		1
Siemens	MD	15	0.20	0.02	2
Siemens	KD	18	0.88	0.10	2
Siemens	KD	20				0.92
Siemens	Primus^a^	10	0.02		1
Siemens	Primus^a^	15	0.12		1
Siemens	Primus^b^	15	0.21		1
Siemens	Primus	15			1	0.20
Elekta	SL‐20	17				0.69
Elekta	SL‐20	18	0.46		1
Elekta	SL‐25	18	0.46		1
Elekta	SL‐25	22				2.37
Elekta	SL‐25	25	1.44	0.31	3
GE	Saturne 41	12				0.24
GE	Saturne 41	15				0.47
GE	Saturne 43	18	1.32		1	1.50
GE	Saturne 43	25				2.40

^a^ With MiMIC device in place.

^b^ With the multileaf collimator set to a $3.8-{\text{cm}}^{2}\times 3.8-{\text{cm}}^{2}$ field.

The activated gold foils were allowed to decay for approximately one day before they were counted on an Eberline model BC‐4 Beta Counter (Eberline Instrument Corp., Santa Fe, NM). The day of decay allowed trace amounts of activated elements such as sodium, to decay so that only the ${\;}^{198}\text{Au}$ emissions were detected. The measured induced activity in each gold foil was a function of the fast neutron fluence rate that was incident on the moderator. The time of irradiation, time elapsed between irradiation, and foil counting, and the count time duration was recorded. Following a procedure described by Palta *et al.* ^1^ and in the American Association of Physicists in Medicine Task Group Report 19,^13^ the count rate per gram for each activated gold foil was related to the fast neutron fluence through a neutron fluence per count rate calibration factor determined for the Eberline Beta counter. The calibration factor was determined by sending several gold foils of known mass to the National Institute of Standards and Technology to be irradiated in a known neutron fluence with an uncertainty of ±2% at the one standard deviation level. These calibration foils were subsequently counted on the Eberline counter to determine the count rate to neutron fluence relationship. The calibration factor used was $3.515\times 10^{6}(n\;{\text{cm}}^{-2}\;\text{gram}\;\text{per}\;\text{counts}\;s^{-1})$.

The total neutron fluence excluding the thermal component was then determined at each point of measurement (points A–E). The measured count rate for each foil and the neutron fluence calibration factor determined for the counting system were used to calculate the fast neutron fluence at each point of measurement.

The total neutron fluence is comprised of three components, thermal, scattered and direct, as indicated in Eq. (1). Solving Eq. (2) for *Q*, excluding the thermal component, yields:

(4) $$Q=\Phi_{\text{dir}+\text{sca}}/(\frac{1}{4\pi d^{2}}+\frac{5.4}{S}).$$

The measured fast neutron fluence for each point of measurement and Eq. (4) were used to determine the neutron source strength for a linac and energy combination. Each *Q* value determined for a linac and energy combination was an average value for all of the measurement points A–E (see Fig. 1). Measurements at isocenter would have resulted in an incorrect elevated neutron source strength value for the linac due to extra photoneutron production in the moderator. None of the measurements of neutron fluence at the isocenter were included in this analysis.

## RESULTS AND DISCUSSION

**Figure 2:**
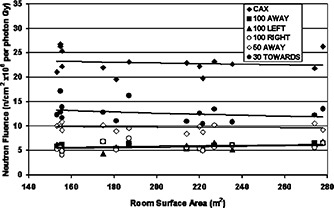
The measured neutron fluence ($n/{\text{cm}}^{2}\times 106$ perphoton Gy) produced by the 18 MV x‐ray beam from 15 Clinac 2100C linear accelerators at six locations in the patient plane as a function of room surface area.

The results of the neutron fluence measurements as a function of room surface area can be seen in Fig. 2. Depending on the location of the measurement point, the magnitude of the neutron fluences differ. However, there is little to no dependence on room surface area. Scatter and thermal neutron fluence components at any point within a treatment vault depend on the room surface area. The data in Fig. 2 suggest that the measured neutron fluence in the patient plane is primarily from the direct neutron fluence originating from the head of the machine and very little contribution is from scattered fast neutrons originating from the rooms walls.

Table I lists the neutron source strengths (*Q*) determined for the linac models and energies measured. The *Q* values (in $10^{12}$ n per photon Gy) range from 0.02 for a Siemens Primus 10 MV beam (with MiMIC device attached) to 1.44 for an Elekta SL25 25 MV x‐ray beam. Table I adds another 13 *Q* values to the data already published.^4,^, ^11^ Many of the *Q* values in Table I are average values for measurements on more than one linac of a specific model and energy. In addition to the *Q* values for the new linac and energy combinations, five linac/energy combinations already had *Q* values listed in the literature.^4,^, ^11^ These five sets of redundant data are in good agreement considering the inherent uncertainty (±20%) in neutron fluence measurements. Apparent exceptions to the good agreement of the results are the relationship between the *Q* values for the Elekta SL25 22 and 25 MV beams, which are $2.37\times 10^{12}$ and $1.44\times 10^{12}$, respectively, and the Philips SL20 17 and 18 MV beam's *Q* values that are $0.69\times 10^{12}$ and $0.46\times 10^{12}$, respectively. There is also a difference between the 24 MV beam from a Clinac 2500 and the 18 MV beam from a Clinac 2100C. The *Q* value for the 2100C 18 MV beam is greater than that for the 24 MV beam and this is best explained by differences in head shielding. In general, for a given linac model, the number of neutrons produced is expected to increase with energy. There are differences in the photon energies, but the change in *Q* is in the opposite direction than would normally be expected. There may also be differences in the head shielding which would explain some of the differences noted. The *Q* value for the SL25 25 MV beam presented in this work represents the average of three linacs and has a standard deviation of $\pm 0.31\times 10^{12}$, whereas the data published by McGinley comes from measurements of one linac which gives one more assurance that the *Q* value from the three accelerators is correct.^4,^, ^10^ The Philips SL20 data (*Q* = 0.46) measured in this work agrees with the measurements for the SL25 (*Q* = 0.46) at the same nominal x‐ray energy. The difference between the 17 and 18 MV beams noted in Table I is most likely attributed to differences in x‐ray energy and uncertainty in the neutron measurements.

Another contribution to the observed differences in *Q* values between accelerators might be due to differences in the head design and shielding. Most current accelerators of the same manufacturer have similar head designs, but subtle changes are made during the manufacturing process and the head design information is considered proprietary by the manufacturers. The subtle changes to the head design probably do not change the neutron production significantly. Some accelerators such as the Clinac 2100C series did undergo a change in head design when the MLC was added.^11^ The *Q* values for the Clinacs with and without MLCs were indistinguishable, indicating no effect on the neutron production even with a substantial change to the machine head. The *Q* values are not significantly different between different models of accelerators from the same manufacturer and energy, however they do differ between accelerators from different manufacturers.

## CONCLUSION

The data in Table I are a compilation of all of the published *Q* values to date. These data will enable physicists to perform the appropriate shielding calculations for most linac/energy combinations following published guidelines for teletherapy shielding.^4^
